# A Novel High-Precision Railway Obstacle Detection Algorithm Based on 3D LiDAR

**DOI:** 10.3390/s24103148

**Published:** 2024-05-15

**Authors:** Zongliang Nan, Guoan Zhu, Xu Zhang, Xuechun Lin, Yingying Yang

**Affiliations:** 1 Laboratory of All-Solid-State Light Sources, Institute of Semiconductors, Chinese Academy of Sciences, Beijing 100083, China; nanzongliang@semi.ac.cn (Z.N.); zhuga@semi.ac.cn (G.Z.); xclin@semi.ac.cn (X.L.); 2 College of Materials Science and Opto-Electronic Technology, University of Chinese Academy of Sciences, Beijing 101407, China; 3 Shenghong (Taizhou) Laser Technology Co., Ltd., Taizhou 318001, China; zhangxuxu@semi.ac.cn

**Keywords:** LiDAR, railway, SFRE, PCA, local-ICP

## Abstract

This article presents a high-precision obstacle detection algorithm using 3D mechanical LiDAR to meet railway safety requirements. To address the potential errors in the point cloud, we propose a calibration method based on projection and a novel rail extraction algorithm that effectively handles terrain variations and preserves the point cloud characteristics of the track area. We address the limitations of the traditional process involving fixed Euclidean thresholds by proposing a modulation function based on directional density variations to adjust the threshold dynamically. Finally, using PCA and local-ICP, we conduct feature analysis and classification of the clustered data to obtain the obstacle clusters. We conducted continuous experiments on the testing site, and the results showed that our system and algorithm achieved an STDR (stable detection rate) of over 95% for obstacles with a size of 15 cm × 15 cm × 15 cm in the range of ±25 m; at the same time, for obstacles of 10 cm × 10 cm × 10 cm, an STDR of over 80% was achieved within a range of ±20 m. This research provides a possible solution and approach for railway security via obstacle detection.

## 1. Introduction

With the rapid advancements in rail transportation in recent years [1,2], integrating fully automated operation modes into railway systems is becoming more prevalent [3]. This necessitates strengthening risk prevention and control measures to avoid collisions with obstacles during rail operations [4]. As a result, there is an urgent demand for detecting potential obstacles along railway tracks, such as pedestrians, animals, and falling rocks. LiDAR technology has emerged as a crucial component in detection compared to traditional visual sensors due to its exceptional obstacle-detection capabilities, precise detection accuracy, and adaptability to different environmental conditions [5,6]. LiDAR collects echo signals from obstacles by utilizing laser pulses, generating a comprehensive three-dimensional point cloud output that provides detailed information about surrounding objects [7,8].

In order to achieve higher detection accuracy, researchers have developed various obstacle-recognition methods specifically designed for point clouds. For instance, deep learning networks such as PointNet [9] and PointNet++ [10] have been introduced for the processing of complex point clouds. These networks utilize fully connected layers to perform tasks like segmentation and classification in intricate scenarios. At the same time, LiDAR obstacle detection algorithms based on CNN networks have been developed and applied [11]. However, machine learning-based methods require many samples and regular updates to the model weights, limiting the timeliness of their application [12].

In contrast, previous non-machine learning algorithms relied on the assumption of flat road surfaces for obstacle identification [13]. For example, Alberto et al. [14] proposed using elevation thresholds derived from consecutive laser returns to differentiate curbside obstacles. Recognizing the limitations of this flat road assumption, Asvadi et al. [15] employed a segmented approach. They utilized the Random Sample Consensus (RANSAC) algorithm for ground point segmentation under various slope conditions and leveraged a voxel grid model to discern stationary and moving obstacle point clouds.

Additionally, other famous techniques have been applied to processing obstacles in point clouds. These include density-based algorithms such as k-means clustering [16], Euclidean clustering [17], and density-based spatial clustering (DSC) [18]. These algorithms aim to group points within a certain threshold around a central point into clusters, which are then used to detect obstacles based on cluster characteristics. However, traditional methods often utilize a fixed neighborhood threshold for the entire point cloud scene. This approach faces limitations because LiDAR point clouds are typically unevenly distributed, with significant density variations.

In addressing this limitation, Gao et al. [19] proposed a dynamic threshold DSC algorithm that overcomes this challenge. Their approach leverages an elliptical model to characterize the local environment and dynamically adapt the neighborhood radius based on the central point’s position. This innovative technique leads to enhanced clustering algorithm performance. It has been successfully applied in obstacle avoidance experiments using onboard LiDAR systems. Jiang et al. [20] enhanced the clustering performance of obstacle clusters by introducing a modulation technique that adjusts the clustering radius based on the horizontal resolution θ and pitch resolution ω of the LiDAR. This modulation enables precise radius adjustments at various locations.

Numerous algorithms using point clouds have been employed in the obstacle detection (OD) process. For instance, Xie et al. [21] successfully detected moving obstacles by integrating a dynamic point-tracking model with a Kalman filter. This approach effectively captures and tracks objects in motion. Similarly, Frank et al. [22] implemented moving object detection in point cloud streaming input by combining the iterative closest point (ICP) algorithm with a Kalman filter, leveraging local convexity criteria.

Before conducting our research, we investigated the application of different types of LiDAR in specific projects. LiDAR has gradually been applied to railway projects, but different applications, such as rail extraction and intersection recognition, rely on different sensor models for the pipeline of the algorithm [23]. B. Borgmann et al. used a Velodyne HDL-64E LiDAR and proposed a height threshold method to achieve segmentation of ground point clouds; the method was based on an implicit shape model (ISM) and successfully achieved detection of person point clouds, but the study did not mention detection of smaller obstacles [24]. P. Burger et al. utilized the scanning characteristics of Velodyne sensors to achieve fast cluster segmentation in off-road environments by labeling discontinuous fixed points. However, this approach relies on Velodyne sensors and is suitable for dynamic point clouds [25]. Current point cloud OD algorithms face challenges such as limited accuracy and suitability in basic scenarios [26]. To overcome these limitations and cater to the obstacle detection requirements in railway applications, we have devised a high-precision OD algorithm using a mechanical 3D LiDAR. Our algorithm offers a comprehensive solution that accurately identifies obstacles in railway environments. We conducted extensive experiments and continuous testing of the developed algorithm within our simulated railway test site, effectively showcasing its remarkable effectiveness and robustness.

**Figure 1:**
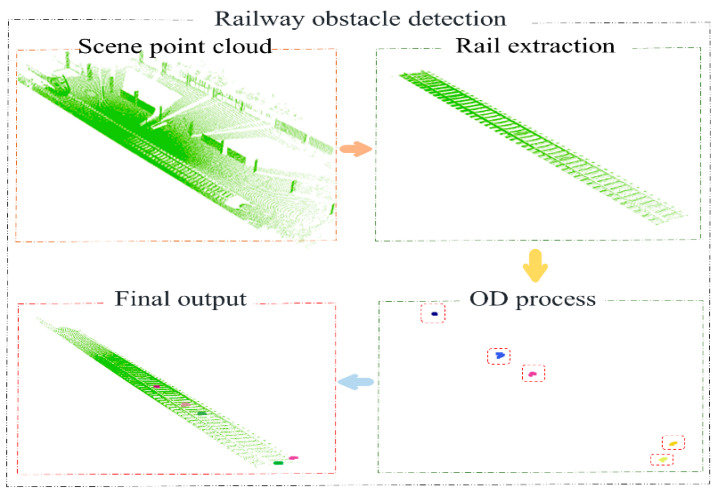
The operational steps of our railway OD algorithm.

This article presents a comprehensive algorithm for railway obstacle detection, as demonstrated in Figure 1. Our contributions are as follows:

(a) We propose a novel method for rail extraction based on LiDAR scanline features. This method overcomes the shortcomings of traditional ground segmentation algorithms in the segmentation process (to effectively filter the point cloud of raised obstacles on the ground and apply the algorithm to less strictly flat road surfaces) and accurately preserves the point cloud information of the track region.
(b) We have addressed the fixed threshold limitation in traditional Euclidean clustering algorithms and proposed an adaptive algorithm with tunable thresholds.
(c) The proposed algorithm achieves an STDR of 96% for obstacles of 15 cm × 15 cm × 15 cm within a range of ±25 m and of 84% for obstacles of 10 cm × 10 cm × 10 cm within a range of ±20 m. We conducted diverse and repeated experiments in a simulated railway environment, yielding satisfactory results and demonstrating the potential for large-scale application.

The starting point of our system is to detect and prevent some dangerous factors (such as falling rocks, mudslides, etc.), and these obstacles invading the track area will affect driving safety. Therefore, our system can be installed in fixed areas with high risk factors to achieve early warning of threatening obstacles.

The workflow of this article is as follows: In Section 2, we introduce the scanning principle of the equipment we used. In Section 3, we introduce the key processes and steps of the algorithm, and we further improve the traditional Euclidean clustering in this study. In Section 4, we conduct performance and robustness testing of the algorithm in the experimental field, and the results demonstrated satisfactory accuracy of the algorithm’s OD performance. Finally, we conclude the article with a summary and outlook.

## 2. Scanning Mechanism of Mechanical LiDAR

**Figure 2:**
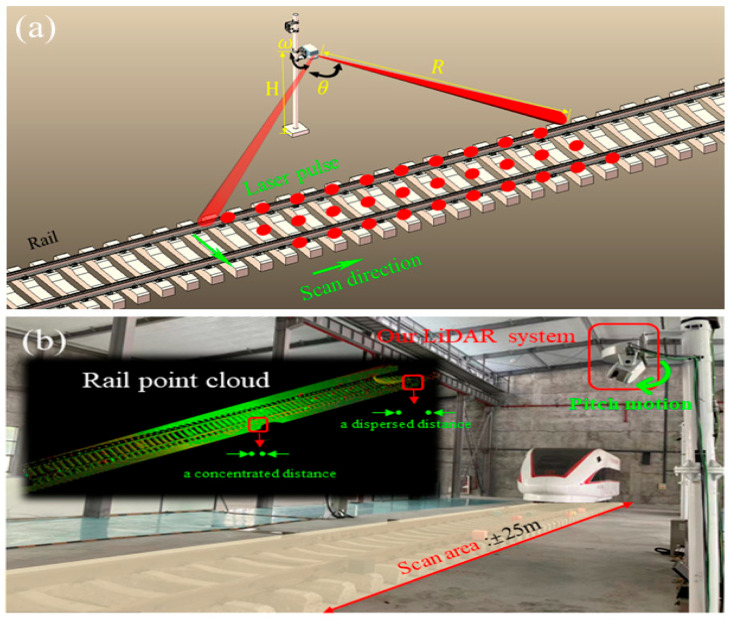
Installation method and scanning mechanism of the 3D mechanical LiDAR. (**a**) is a schematic diagram that we use to describe the scanning method of the device; (**b**) is a schematic diagram of installing our equipment on site, with the equipment installed in a fixed position to achieve scanning within a 50m range of the track area.

The 3D LiDAR depicted in Figure 2 is utilized within our study. While the system’s autonomous development falls outside this article’s scope, our primary focus is elucidating its scanning mechanism. Our device completes the acquisition of a single scanning line in the horizontal direction with a frequency f. The pulse points are distributed according to a fixed lateral angle resolution, and high-precision servo motors are used to control the pitch direction. The scanning line is distributed according to a fixed pitch angle resolution, which is used to obtain three-dimensional point cloud data for the rail area within the range of ±25 m. We control the maximum error of scanning for the same point to be below 2 cm. Due to our scanning mechanism, we obtained a point cloud map with a concentrated distance between points in the central region and a dispersed distance between points in the edge region (Figure 2b).

We exercise control over the precision of the point cloud map by adjusting the scanning time T and the pitch scanning range [$\omega_{start},\omega_{stop}$]. The following formula is used to calculated the parameters:

(1) k=T×f

(2) $$\phi =\omega_{stop}-\omega_{start}$$

(3) $$\delta_{pitch}=\phi \;/\;k$$

In Equations (1)–(3), k represents the number of scanning lines in a single frame of the point cloud map, T represents the scanning time, $\delta_{horizontal}$ represents the known horizontal angular resolution, and $\delta_{pitch}$ signifies the pitch angular resolution.

## 3. Implementation of the OD Algorithm

In this section, we provide a comprehensive description of our algorithm’s framework. We begin with the point cloud calibration process and then proceed to explain the rail extraction method using both background point cloud (BP) and foreground point cloud (FP) techniques to achieve OD. Each step will be elaborated on in detail.

**Figure 3:**
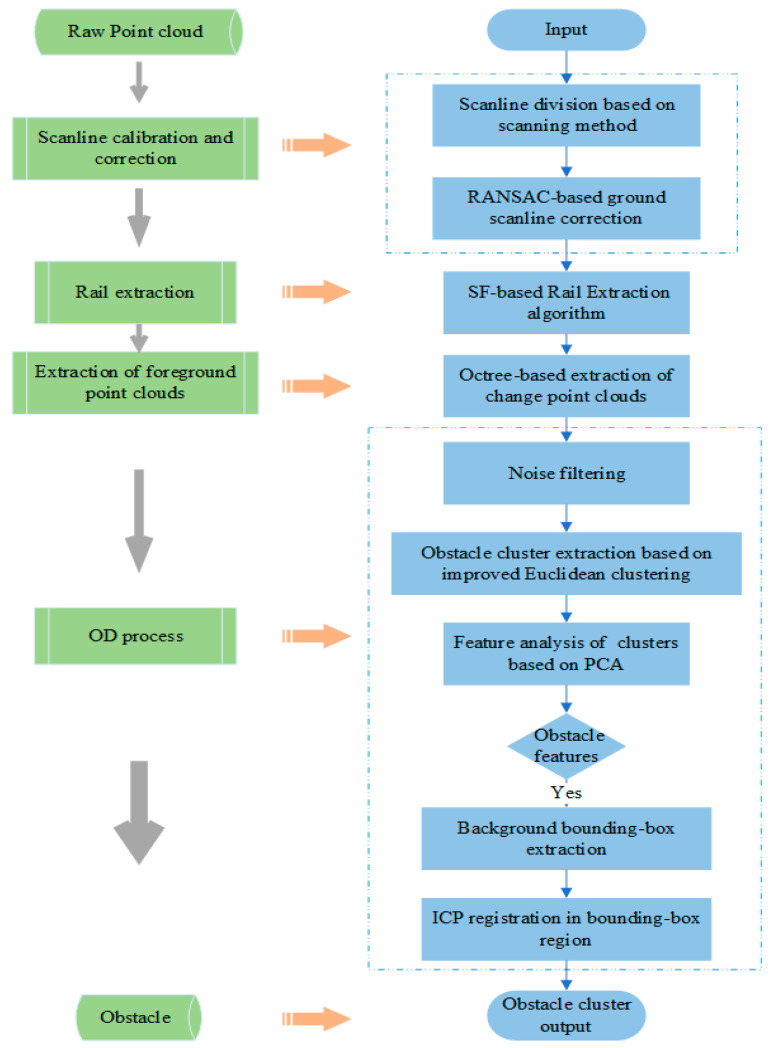
The key steps of our algorithm and its corresponding flowchart.

The algorithm framework diagram, depicted in Figure 3, illustrates the sequential processing flow.

We have implemented error correction techniques to global scanning lines within a single frame of the point cloud map for both BP and FP. Additionally, we introduce our track extraction algorithm, SFRE (scanline feature-based rail extraction), which leverages the distinct characteristics of scanning lines.

Once the track extraction process is completed, we apply Octree downsampling to the FP, reducing computational complexity. In the primary OD stage, we employ essential algorithms such as Euclidean clustering, PCA (principal component analysis), and local-ICP (local iterative closest point) to perform detailed feature analysis on point cloud clusters. Furthermore, we propose a tunable threshold Euclidean clustering algorithm to address traditional Euclidean methods’ limitations when applied to non-uniformly spaced point clouds. This method effectively identifies and outputs point cloud clusters corresponding to obstacles.

### 3.1. Scanline Calibration and Correction

Our system ensures that point cloud data adhere to a regular single-line distribution, typically containing between 150 k and 200 k points per scene (based on Equation (4)). However, practical applications may introduce errors, such as point cloud drift, due to installation errors and natural factors like pole sway caused by strong winds. Accurate correction of point cloud errors is crucial as it significantly affects processes like filtering and registration. We first correct the point cloud errors based on the scanlines’ distribution characteristics to address this.

We can obtain the transformation formula for coordinates (*x*, *y*, *z*) based on the raw pulse distance *R* and device height *H* [27].

(4) n=m ∗ T/f

(5) $$\left\{\begin{array}{c} x=R\;*\;sin\omega \;*\;cos\theta \;\;\;\;\;\;\;\;\\ \;\;y=R\;*\;sin\theta \;\;\;\;\;\;\;\;\;\;\;\;\;\;\;\;\;\;\;\;\;\;\;\\ z=H-R\;*\;cos\omega \;*\;cos\theta \end{array}\right.$$

In Equation (4), n represents the total number of points in one frame, m represents the number of scanning lines, T represents the scanning time of a single frame, and f represents the frequency of a single line. In Equation (5), θ represents the horizontal angle of the pulse, ω represents the pitch angle of the pulse, H represents the device’s hanging height, and R represents the distance of the pulse. 

**Figure 4:**
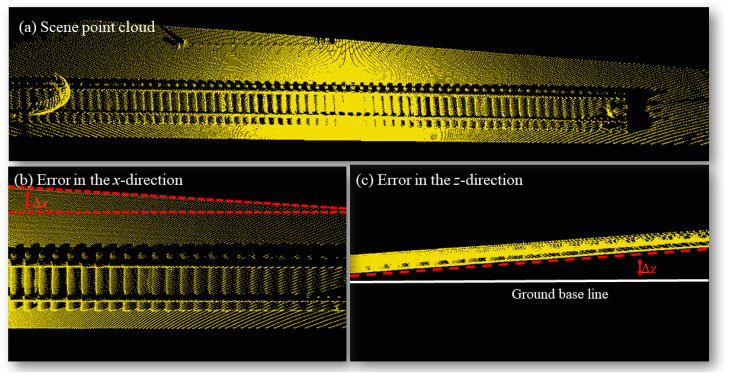
Scene point cloud map and two primary sources of point cloud errors: Δx caused by pitch motion and Δz caused by installation.

During the scanning process, the motor’s continuous variation results in each pulse point deviating from the ideal *ω* value. Factors such as shaking and installation introduce errors that accumulate over time, leading to drift in the point cloud of the scene (as depicted in Figure 4). To tackle this issue effectively, we propose a specialized global error correction method.

**Figure 5:**
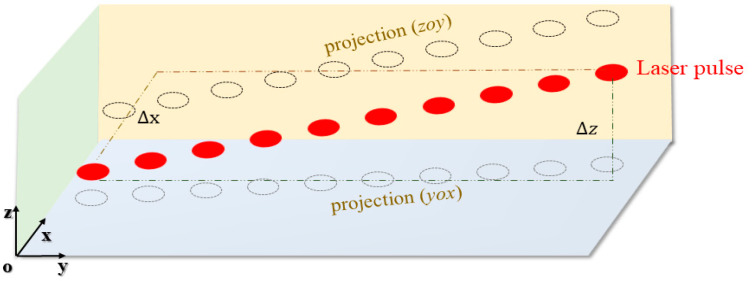
Scanning line projection mechanism in error correction process.

The variable Max Δz represents the deviation of the scanning lines in the *z*-direction, which is driven by external factors. The variable Max Δx represents the error caused by the same scanning line, which does not follow the same ω value in the *x*-direction. In our SFRE algorithm, we rely on the *x*-distribution of the scanning lines. Therefore, we employ the following steps to correct the point cloud (Figure 5):

(a) We input the set of points *P* from the entire point cloud. Given the known scanning frequency *f*, we can determine the number of points *m* in a single scanning line. Using Equation (5), we can accurately segment the input point cloud into individual scanning lines.

(6) $$I_{i}=\left\{P_{j}|P_{j}\epsilon P_{\left[m\;*\;i,m\;*\;\left(i+1\right)\right]}\right\}(I_{i}\epsilon I,0<i\leq k)$$

where I represents the clustered set of scan lines we have divided and k represents the maximum number of scan lines in a single frame of our point cloud.

(b) We randomly select a baseline scanline, project it onto the *xoy* plane, and choose a set of scan points. By using the RANSAC algorithm to fit a line [28], we can obtain the spatial parameters of the scanline. Then, we use it to obtain the deviation angle. Similarly, projecting onto the *yoz* plane gives us the deviation angle. These angles are used to correct the errors caused in Figure 4.

(7) ax+by+cz+d=0

(c) We can obtain the deviation angle based on the fitting parameters of the scanline. We utilize angles $\alpha_{xoy}$ and $\alpha_{yoz}$ to construct the corrective rotation matrix *R*_1_ and *R*_2_ for the scene point cloud representation:

(8) $$R_{1}=\left|\begin{array}{ccc} 1 & 0 & 0\\ 0 & \cos(\alpha_{xoy}) & -\sin(\alpha_{xoy})\\ 0 & \sin(\alpha_{xoy}) & \cos(\alpha_{xoy}) \end{array}\right|$$

(9) $$R_{2}=\left|\begin{array}{ccc} 1 & 0 & 0\\ 0 & \cos(\alpha_{yoz}) & -\sin(\alpha_{yoz})\\ 0 & \sin(\alpha_{yoz}) & \cos(\alpha_{yoz}) \end{array}\right|$$

(10) $$P^{\prime }=R_{1}R_{2}P$$

### 3.2. Railway Extraction

When dealing with large-scale ground point cloud processing, traditional methods for ground segmentation often employ algorithms like RANSAC [29]. However, dealing with the rail track recognition for OD in specific areas of railways, traditional ground segmentation methods encounter significant challenges due to the varying terrains and undulations [30]. Consequently, existing ground segmentation algorithms primarily rely on the assumption of flat ground, resulting in decreased robustness and applicability when confronted with natural terrain variations, including slopes and undulations.

To address this issue, we propose a rail track extraction algorithm based on the scanning characteristics of LiDAR. By analyzing the distribution characteristics of scan lines, our method aims to improve the accuracy and reliability of rail track recognition in the presence of diverse terrains and undulations.

**Figure 6:**
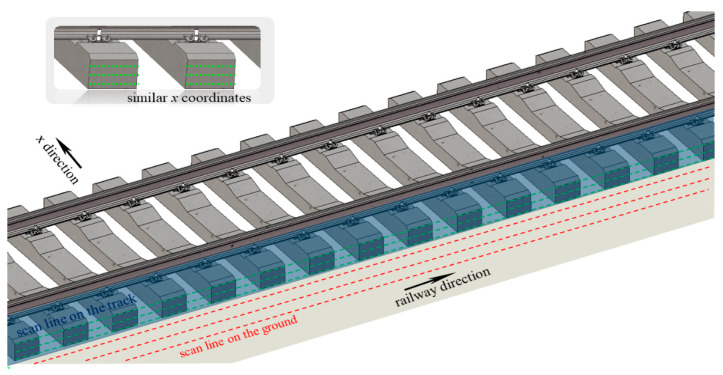
The scanning process of the scanline on the railway track.

As shown in Figure 6, it is evident that after conducting overall calibration and calibration specifically in the *x*-direction on the point cloud data, a noticeable “enrichment” phenomenon can be observed in the scan lines during the scanning process from the baseline to the rail track. This implies that the point cloud data yield valuable information in the *x*-direction during the scanning transformation from the baseline to the rail track. We segmented the scanlines in previous steps and calibrated the point cloud data. Leveraging the features we have discovered, we utilize the enriched region of the *x*-coordinate as the track boundary for straight-through filtering, enabling us to obtain precise point cloud data about the rail track.

(11) $$p_{rail}\epsilon P\left(x^{,}\leq p_{rail}\cdot x\leq x^{,,}\right)$$

The pseudo-code for the essential parts is shown as Algorithm 1:
**Algorithm 1:** Railway scanline division and calibration, SFRE process**Input:** point cloud set P, the number of pulses of a scanning line m;**Output:** Rail point cloud
$P_{rail}$
1: k=0;2: for i=1; i<n; i++ **do**3:   Line cluster I(k) ← m points
4:    k++
5: **end for** 6: I(g) ← ground scanning line 7: p·z=0 (p ∈ I(g)) 8: $\alpha_{xoy}\;\leftarrow \;I(g)[50,\;m-50]\;$RANSAC line fitting 9: $R_{1}\;\leftarrow \;\alpha_{xoy}$ 10: p·x=0 (p ∈ I(g)) 11: $\alpha_{yoz}\;\leftarrow \;I(g)[50,\;m-50]\;$RANSAC line fitting 12: $R_{2}\;\leftarrow \;\alpha_{yoz}$ 13: $P\;\prime =R_{1}R_{2}P$ 14:  bucket<key, value> 15: **for** i=0; i<P′·size(); i++**do** 16:  **if**
bucket·count(P′·x/A)>0 **then** 17:      bucket[P′·x/A]++ 18:  **else** bucket[P′·x/A]=1 19:  **end if** 20: **end for** 21: //Get the the two keys with the maximum values 22: key1, key2 ← Max(bucket[key1]), Max(bucket[key2]) 23: $P_{rail}\cdot x1=key1\;*\;A$ 24: $P_{rail}\cdot x2=key2\;*\;A$ 25: //f(P, x1, x2) filtering in the x direction with set P 26: $Prail\;\leftarrow \;f(P\;\prime ,\;P_{rail}\cdot x1,\;P_{rail}\cdot x2)$ 27: return Prail

### 3.3. OD Process

Before entering the OD phase, we utilized Octree to reduce computational complexity and minimize computational overhead [31]. We downsampled using a fixed step size ρ, obtaining sparse samples of the original point cloud for subsequent calculations.

#### 3.3.1. Improved Euclidean Clustering

**Figure 7:**
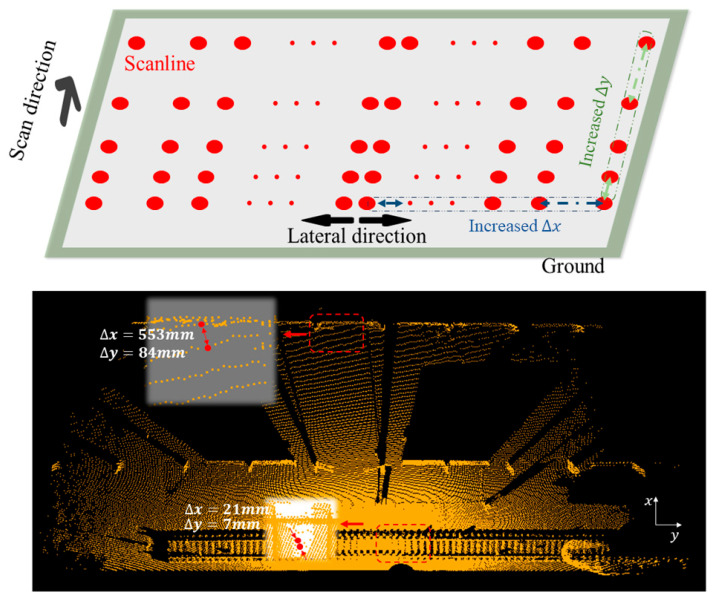
The distribution trend of point cloud density in the *x-* and *y*-directions within the actual scanning scene, where Δx represents the distance between the *x*-directions of two points on the same plane and Δy represents the distance between the *y*-directions of two points [33].

The traditional Euclidean clustering algorithm is implemented through the following steps: Select a center point *q_1_*, search for *n* nearest points within a specified threshold value, and create a set *P* that satisfies the threshold. Next, select another point *q_2_* from set *P* and repeat the process until a complete set *P* is formed. The traditional Euclidean clustering algorithm [32] uses a fixed threshold value throughout the process. However, in practical applications (as shown in Figure 7), the point cloud density varies significantly with distance *R* along horizontal and vertical scanning directions. This variation reduces the adaptability of the traditional Euclidean clustering algorithm. To address this issue, we propose a tunable threshold Euclidean clustering method.

We observed that the region with the highest point cloud density occurs in the central area of the scanning line at the starting pitch angle ω. Therefore, we propose the following tunable threshold strategy:

(12) $$\varepsilon_{adaptive}=f\left(x,y,p_{i}\right)\varepsilon$$

In this equation, the variable ε represents the initial input threshold in traditional Euclidean clustering algorithms. Additionally, $f\left(x,y,p_{i}\right)$ signifies the modulation function associated with the *x*- and *y*-directions of the clustering center point *p_i_*. The detailed form of the modulation function can be found in Section 4.

#### 3.3.2. PCA Process for Clusters

After obtaining different point cloud clusters using the improved Euclidean clustering method, we performed principal component analysis (PCA) [34] on the point cloud to obtain feature information of obstacle clusters O for filtering and differentiation. The PCA process is as follows [35]: we input the obstacle clusters according to the index and obtain the number of points in the *i*-th cluster, denoted as *O_i_*. If the information in the point cloud is (*x, y, z*), we obtain an *n ×* 3 point cloud matrix ξ. We perform mean normalization on the data of each point in matrix ξ, and then obtain the covariance matrix *C*; using Equation (16), we obtain the three eigenvectors $E(e_{1},e_{2},e_{3})$ and eigenvalues $\left(\lambda_{1},\lambda_{2},\lambda_{3}\right)$, where Λ is a diagonal matrix.

(13) $$O_{s}=\frac{1}{n}\sum_{i=1}^{n}p_{i\;}{\;(p}_{i\;}\epsilon \xi )$$

(14) $$p_{i}=p_{i}-O_{s}\;(i\;\epsilon \;[1,n])$$

(15) $$C=\frac{1}{\;n}\xi \xi^{T}$$

(16) $$E^{T}CE=\Lambda$$

**Figure 8:**
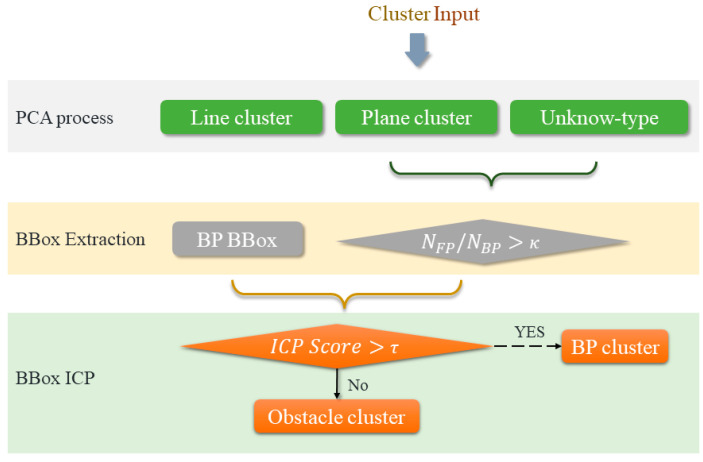
The mechanisms and procedures of PCA and local-ICP processing in the OD process.

We arrange the eigenvalues in descending order as $\lambda_{1}>\lambda_{2}>\lambda_{3}$. According to the relationship between eigenvalues, we make the following division of cluster types: line type, plane type, and unknown type (Figure 8).

(17) $$\left\{\begin{array}{c} \lambda_{1}/\lambda_{2}>\mu_{1}\;lines\\ \;else\;\left\{\begin{array}{c} \lambda_{2}/\lambda_{3}>\mu_{2}\\ unknow \end{array}\right. \end{array}\;plane\right.$$

We obtained a collection of multiple clusters, denoted as ${Τ}_{1},{Τ}_{2}$ and ${Τ}_{3}$. Where ${Τ}_{1}$ represents a cluster collection of lines point clouds, ${Τ}_{2}$ represents a cluster collection of plane point clouds, and ${Τ}_{3}$ represents a cluster collection of unknown point clouds. By default, we only remove cluster ${Τ}_{1}$. For cluster ${Τ}_{2}$, sometimes due to the perspective, we can only see one side of the object. Therefore, it is treated as a potential obstacle cluster. We extract equal-sized regions from the background point cloud based on the indices of all clusters. When Formula (18) is satisfied, we consider that the cluster is more likely to be an obstacle.

(18) $$N_{FP}/N_{BP}>\kappa$$

Here, $N_{BP}$ represents the number of point clouds within the bounding box belonging to BP, $N_{FP}$ represents the number of point clouds within the bounding box belonging to FP, and κ is our parameter threshold.

#### 3.3.3. Local-ICP Process for Clusters

The ICP process is commonly used for point cloud registration, aiming to find the best correspondence between a source point cloud and a target point cloud through iterative refinement [36]. Once we obtain the bounding boxes of the BP (BP BBox) and FP (FP BBox), we can achieve the alignment of the two through the following steps using local-ICP [34]:

(a) Use the BP BBox point cloud as the source input point cloud set *P* and the FP BBox point cloud as the target point cloud set *Q*.
(b) Find the correspondence point *q_i_* for each *p_i_*.
(c) Use an energy minimization strategy to find the optimal transformation matrix *T* (*R*, *t*) that satisfies Equation (19).
(d) Repeat the iteration process until step (c) converges to meet the threshold.

(19) $$f\left(R,t\right)=\underset{R,t}{\min}\sum_{i=1}^{n}{\left\|Rp_{i}+t-q_{i}\right\|}^{2}$$

After registration using local-ICP, when the registration score is high, the probability of the cluster not belonging to obstacles is high. Taking the complement of the cluster set results in the obstacle set.

(20) $$\tau =\frac{\sum_{i=1}^{n}{\left\|p_{i}-q_{i}\right\|}^{2}}{n}$$

## 4. Experiment

To evaluate the effectiveness of our proposed algorithm, we conducted a quantitative analysis by creating a standardized simulated railway track measuring 50 m in length. The algorithm was subsequently deployed in our experimental site, and this section provides a detailed demonstration of our algorithm’s strategy.

### 4.1. Evaluation of Error Correction

We refer to Table 1 for the hardware parameters of the equipment used. In order to address the errors mentioned in Section 2, we partitioned the scan lines based on a fixed number of points per scanline after obtaining the complete point cloud of a single frame scene. For calibration of these two types of errors, we applied Formula (21):

(21) $$\left\{\begin{array}{c} \Delta x=p_{i}\cdot x-p_{j}\cdot x\;\;\;\;\;\;\;\;\;\;\;\;\;\;\;\;\;\;\;\;\;\;\;\;\;\;\;\;\\ \Delta z=p_{i}\cdot z-p_{j}\cdot z\;\;(p_{i},p_{j}\epsilon \;p\subset I) \end{array}\right.$$

**Figure 9:**
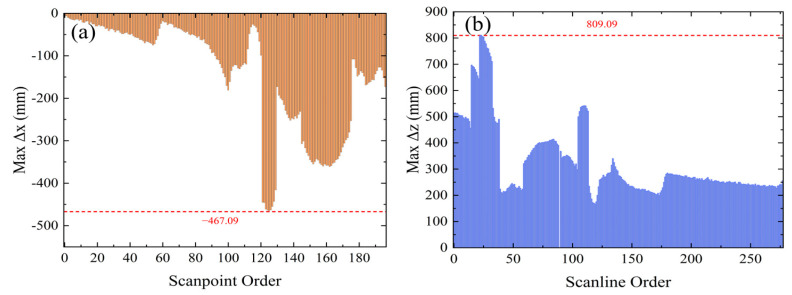
(**a**) Maximum statistical error of Δx in random scene point clouds; (**b**) maximum statistical error of Δz in random scene point clouds.

The variables $p_{i}$ and $p_{j}$ represent the *i*-th and *j*-th scanned points in the scanline p (belonging to scan line clusters I). Given the high probability of noise near the edges of scan lines and their high variability, we measured *x* and *z* using *i* = 60 and *j* = 350. We statistically analyzed the maximum error of all scan lines in random scenes (Figure 9), revealing that the maximum values of x and z were 467 mm and 809 mm. This result confirms the effectiveness of our correction strategy. Furthermore, to obtain point cloud information with accurate coordinates for subsequent algorithmic steps, we calibrated the point cloud data using the fitted matrix *R*_1_ and *R*_2_.

### 4.2. Evaluation of SFRE Method

**Figure 10:**
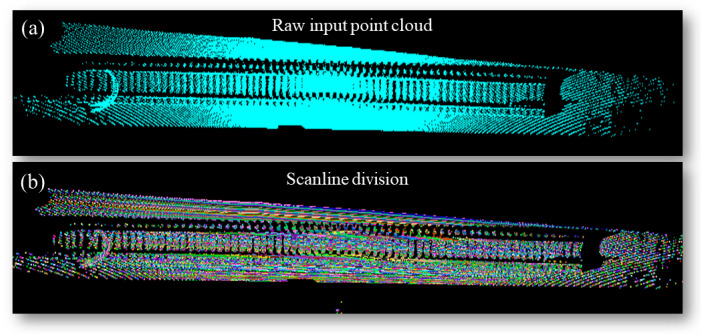
(**a**) Raw input point cloud; (**b**) scanline division process, and we used the same color for each scanning line.

**Figure 11:**
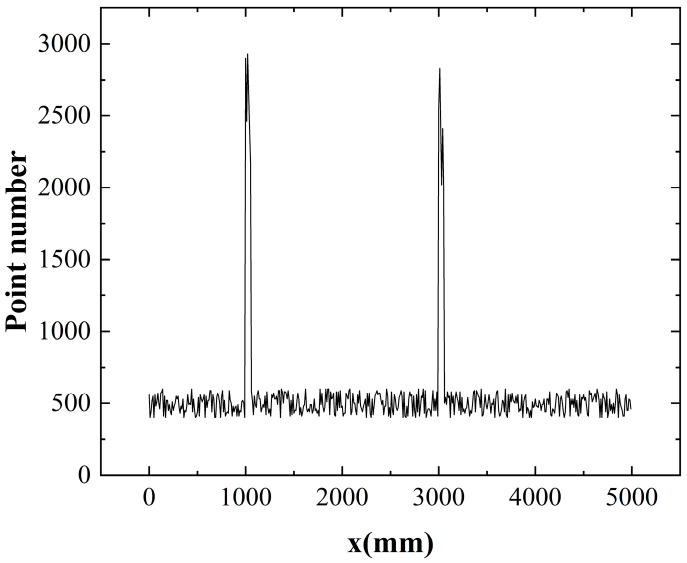
Statistical analysis of the number of scan points at the same *x* position obtained using Formula (22).

We analyzed the *x*-coordinate of the scan line set *I*, as depicted in Figure 10. During the scanning process from the ground base to the track, we observed a notable clustering of *x*-coordinates within a narrow range of variations. This trend is illustrated by the pattern shown in Figure 11. Leveraging this observation, we can identify abrupt changes in the *x*-coordinate as indicative of the boundary regions of the track. More specifically, when the condition specified by Formula (22) is satisfied, it indicates the presence of an edge region in the track.

(22) ∆m/∆p·x>A(p⊂I)

Here, *I* represents the set of clusters of scanlines and *p* is a subset of this set. The formula indicates that the region with a sudden change in the pulse count corresponds to the edge region of the track, as the *x*-coordinate between scan lines changes.

We compared our developed track extraction algorithm, the traditional RANSAC ground segmentation, and the region growing algorithm based on the point cloud library (PCL). It is essential to strike a balance in segmentation, as excessive segmentation can lead to losing track features within the detected region. In contrast, insufficient segmentation may result in point cloud outliers being mistakenly classified as noise. To evaluate their performance, we meticulously selected random scenes and measured the runtime of each algorithm, as well as the retention rate of valid points.

(23) $$RR=\frac{N(P_{rail})}{N(P)}$$

where $N(P_{rail})$ refers to the number of point clouds in the segmented track area, while N(P) refers to the total number of point clouds.

**Figure 12:**
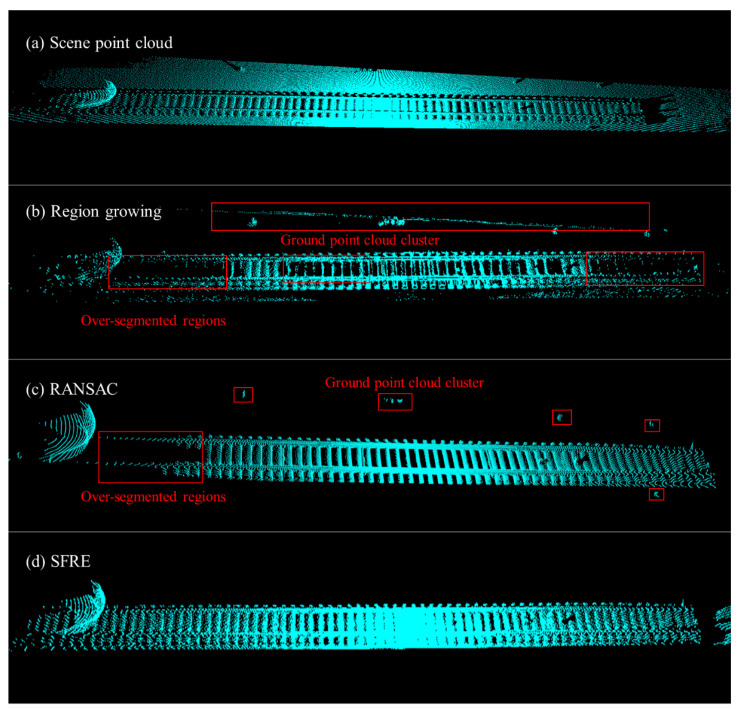
A comparison of the segmentation and extraction results from three algorithms: (**a**) scene point cloud; (**b**) ground segmentation using the region growing method (neighbor point: 10; smoothing threshold: 3 rad; curvature threshold: 1 rad); (**c**) ground segmentation using RANSAC (iterations: 1500; distance threshold: 85 mm); (**d**) track extraction result using our SFRE algorithm.

**Figure 13:**
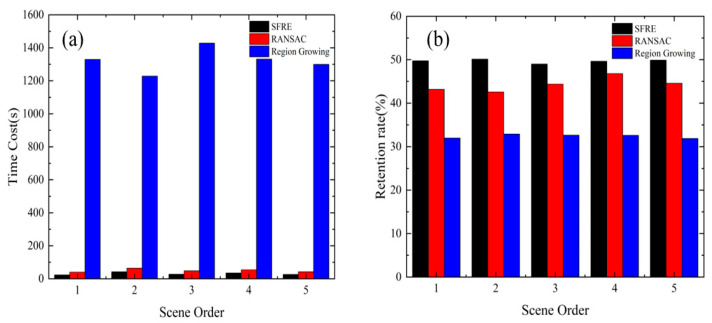
A comparison of three algorithms: RANSAC ground segmentation, region growing segmentation, and SFRE. The figure presents two key aspects: (**a**) shows the computational time required by each algorithm, and (**b**) shows the retention rate of valid points.

As depicted in Figure 12 after adjusting to the optimal parameters, both the RANSAC algorithm and the region growing algorithm exhibited varying degrees of over-segmentation and under-segmentation, with non-ground clusters unable to be fully separated. Our algorithm showcases remarkable robustness when handling minor terrain variations or small-scale, irregularly shaped objects on the ground. It can accurately extract the track while effectively filtering out interfering point clouds from non-detection areas. Furthermore, in Figure 13, our algorithm demonstrates high computational efficiency in terms of runtime, enabling the preservation of a more significant number of feature point clouds in the track region. This facilitates seamless progress in subsequent analysis steps.

### 4.3. Evaluation of Improved Euclidean Clustering

We conducted a quantitative analysis of the distribution difference in the *x*- and *y*-directions based on the scanning characteristics of the device.

(24) $$\left\{\begin{array}{c} x_{i}=\frac{H}{\cos\omega }\;*\;tan\left|\theta_{i}\right|\;({0\leq \theta }_{i}<\frac{\pi }{2})\\ \theta_{i}=i\;*\;\delta_{horizontal}\;\left\{\left.i|0\leq i<\frac{m}{2}\right\}\right.\\ ∆x=x_{i+1}-x_{i} \end{array}\right.$$

(25) $$\left\{\begin{array}{c} y_{j}=H\;*\;tan\omega_{j}\;(\omega_{0}\leq \omega_{j}\leq \omega_{t})\\ \omega_{j}=j\;*\;\delta_{pitch}\;\left\{\left.j|1\leq i\leq k\right\}\right.\\ ∆y=y_{j+1}-y_{j} \end{array}\right.$$

**Figure 14:**
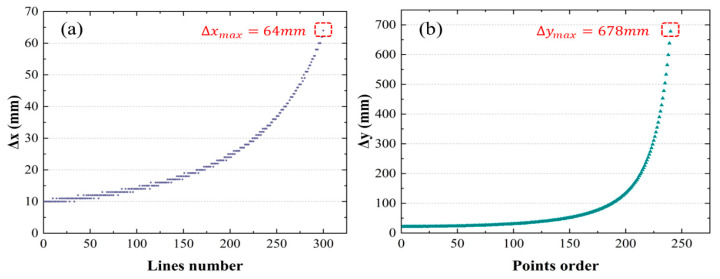
Statistical analysis of ∆x and ∆y in the *x-* and *y*-directions, calculated using Formulas (24) and (25).

Using Equations (24) and (25), we have chosen the parameters ∆*x* and ∆*y* to represent the density distribution trends of the point cloud data along the *x* and *y* directions. Our aim is to ensure that these variables accurately represent the graphical representation of the point cloud’s density distribution (the results are shown in Figure 14).

(26) $$f\left(x,y\right)=e^{\frac{\left|p_{i}\cdot x\right|}{x_{max}}+\frac{\left|p_{i}\cdot y\right|}{y_{max}}+1}$$

Considering the exponential distribution of spacing differences in the *x-* and *y*-directions and aiming to limit threshold divergence, we have employed Formula (26) as a modulation function. In this formula, $p_{i}\cdot x$ represents the *x*-coordinate of the iterative center point $p_{i}$, $p_{i}\cdot y$ represents the *y*-coordinate of the center point $p_{i}$, and *x_max_* and *y_max_* represent the boundary values of *x* and *y* after removing outliers. By dynamically adjusting the clustering centroid points $p_{i}$, our algorithm achieves tunable threshold control in both the *x* and *y* directions.

**Figure 15:**
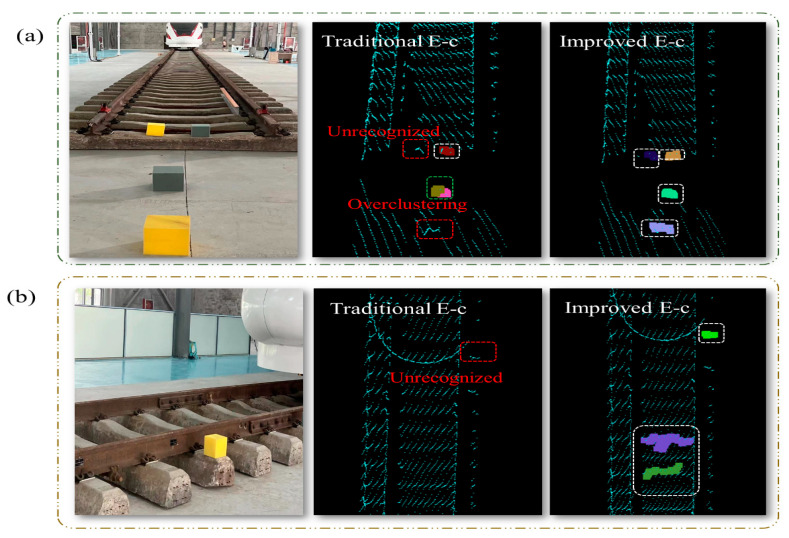
Application analysis of traditional Euclidean algorithm and improved Euclidean algorithm for long-range distances: (**a**) clustering implementation in multi-box scenario (*y* = −2500 mm); (**b**) clustering effect for small objects at long distances (*y* = 2000 mm).

We conducted extensive experimental scenarios to evaluate our modulation function’s effectiveness. The results demonstrated that suspicious clusters were effectively detected even in distant areas, minimizing the risk of missing small objects at far distances (as shown in Figure 15). Moreover, it significantly reduced the occurrence of secondary splitting within the same cluster due to distance divergence caused by LiDAR at far distances. This exemplifies the high detection rate of our algorithm, particularly for high-precision objects.

Overall, our modulation function enables precise and adaptable threshold control, ensuring reliable object detection and reducing the impact of distance divergence on clustering outcomes.

### 4.4. OD Process Based on PCA and Local-ICP

After identifying a cluster of suspicious obstacles O, which may contain numerous false positives ($O_{line}\cup O_{plane}\cup O_{else}=O$). We applied PCA to the clusters to extract a subset that excludes the false obstacle clusters $O_{line}$. This step allowed us to categorize the point cloud clusters into plane and unknown point clouds. Subsequently, we extracted the point clouds in the BP BBox for these clusters and performed sequential registration using local-ICP. To further refine the results, we employed Formula (19) to enhance the filtering of the obstacle set, resulting in $O_{obstacles}$ ($O_{obstacles}\subseteq {(O}_{plane}\cup O_{else})$). Throughout this process, we fine-tuned the parameters and conducted experiments to optimize the outcome.

To evaluate the performance of our algorithm, we conducted performance tests on the experimental site using the parameters listed in Table 2. The stable results obtained from these tests are documented in Table 3, where we primarily utilized the SIDR (single detection rate) and STDR (stable detection rate) as our detection metrics.

(27) $$SIDR=\frac{S_{single}}{S}$$

(28) $$STDR=\frac{S_{stable}}{S}$$

where *S* represents the total number of samples, $S_{single}$ represents the number of samples detected in a single instance, and $S_{stable}$ represents the number of samples detected more than once.

**Figure 16:**
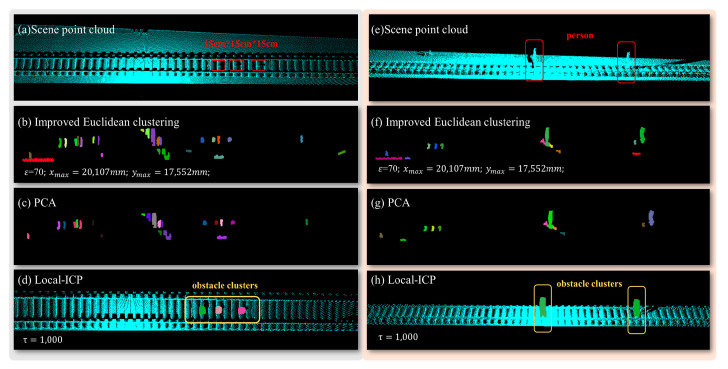
The main process and diagrams of the OD algorithm: (**a**) multiple box obstacles with dimensions 15 cm × 15 cm × 15 cm; (**b**) cluster *O* after the improved Euclidean algorithm is applied (${\varepsilon =70;x}_{max}=20107\;mm;y_{max}=17552\;mm$); (**c**) the collection of obstacle clusters obtained after performing PCA; (**d**) the correct cluster $O_{obstacles}$ achieved by using the local-ICP algorithm (τ=1000); (**e**–**h**) a comparable processing procedure for obstacles represented as human entities.

Extensive test results have demonstrated that our algorithm exhibits sufficient recognition capability for obstacles of size 15 cm × 15 cm × 15 cm at 25 m on both sides of the track. Additionally, it also demonstrates the ability to recognize obstacles of size 10 cm × 10 cm × 10 cm at 20 m on both sides (Figure 16 is a step-by-step diagram of our algorithm pipeline running). We have also compared the indicators of similar algorithms that have been reported and listed them in Table 4. Currently, our algorithm has shown better performance in terms of its detection size with the obstacles, tolerance for obstacle size, and detection stability.

**Figure 17:**
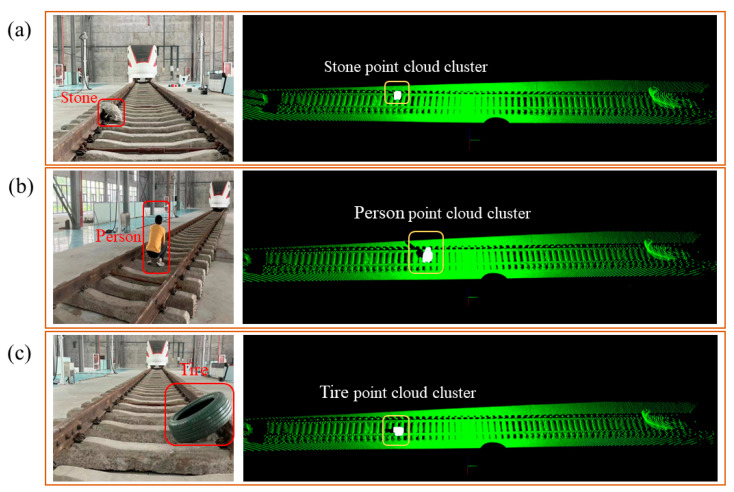
We additionally placed three types of irregular obstacles and displayed on-site photos and algorithm operation results: (**a**) an irregular stone; (**b**) A person located in the track area; (**c**) a tire located in the track area. All types of obstacles have been successfully detected.

In addition, during the process of train transportation, some threatening obstacles often appear in the form of irregular obstacles. To further demonstrate the detection effect of our algorithm on irregular obstacles, we tested the algorithm on large-sized stones, pedestrians, and other obstacles that may affect train operation. We adopted the same testing parameters in Table 2 and obtained the algorithm running results as shown in Figure 17. The results show that our algorithm has successfully detected all possible sample obstacles at present and that our system provides a new solution for railway safety.

## 5. Conclusions

The application of LiDAR in railway OD holds significant research potential. Using the 3D mechanical LiDAR, we have developed an innovative algorithm for obstacle detection based on track area point clouds.

Firstly, we analyzed and corrected the sources of error causing overall misalignment in the point cloud data. By calibrating the point cloud data, we achieved improved accuracy. Instead of relying on traditional ground segmentation algorithms, we employed an SFRE algorithm that retains the track characteristics more effectively. Additionally, we applied Octree downsampling to reduce computational overhead.

During the Euclidean clustering process, we encountered limitations with fixed-threshold applications. To address this, we introduced a modulation function based on the distribution of point cloud density, enabling adaptive neighborhood adjustment. The results demonstrated the effectiveness of our clustering approach, even when dealing with reduced point cloud density.

Following extracting Euclidean clusters from the point cloud data, we implemented PCA to identify potential obstacle sets. Using local-ICP, we filtered out false positive clusters that exhibited significant differences compared to the background point clouds, accurately identifying obstacle point clouds. To ensure robustness, we constructed a standardized railway simulation site to test and optimize our algorithm continuously. The results demonstrated stable detection capability for 10 cm × 10 cm × 10 cm obstacles at 20 m on both sides of the track, with accuracy thoroughly evaluated.

In future efforts, we will further optimize our algorithm to ensure its robust operation under challenging weather conditions such as heavy fog and intense rainfall.

## Figures and Tables

**Table 1:** Hardware parameters of the equipment.

Variable	Definition	Value
f	Single line frequency	50 (Hz)
m	Number of points in a single scan line	511
T	Scan time	6 (*s*)
H	Device height	3200 (mm)
$\delta_{pitch}$	Pitch angle resolution	0.13(°)
$\delta_{horizontal}$	Horizontal angular resolution	0.33(°)
k	Scan line number	300

**Table 2:** Parameter values of the key steps in our algorithm workflow.

ρ	ε	$\mu_{1}$	$\mu_{2}$	κ	τ
100	70	100	10	1.1	1000

**Table 3:** After conducting a substantial number of sample experiments, we evaluated the performance of our algorithm using the SIDR and STDR metrics.

Obstacles	Size (cm)	Detection Distance (m)							
		0		10		20		25	
		SIDR	STDR	SIDR	STDR	SIDR	STDR	SIDR	STDR
person	50 ×50 × 175	100%	100%	100%	100%	100%	100%	100%	100%
box	20 × 20 × 20	100%	100%	100%	100%	100%	100%	100%	100%
box	15 × 15 × 15	100%	100%	100%	100%	95%	100%	92%	96%
box	10 × 10 × 10	93%	97%	86%	94%	72%	84%	-	-

**Table 4:** Comparison of metrics between our model and similar algorithms.

Model	Min Detection Size (cm)	Max Range of Action (m)	Stable Detection Rate (%)
Vitor [37]	30	10	-
J. Li [38]	10	<15	-
G. Zhu [39]	10	20	>70
Ours	10	20	>84

## Data Availability

The authors confirm that the data supporting the findings of this study are available within the article.
